# Reference effect measures for quantifying, comparing and visualizing variation from random and fixed effects in non-normal multilevel models, with applications to site variation in medical procedure use and outcomes

**DOI:** 10.1186/s12874-018-0517-7

**Published:** 2018-07-06

**Authors:** Thomas J. Glorioso, Gary K. Grunwald, P. Michael Ho, Thomas M. Maddox

**Affiliations:** 1 VA Eastern Colorado Health Care System, 13611 E. Colfax Ave, A151, Aurora, Denver, CO 80045 USA; 2 Department of Biostatistics and Informatics, Colorado School of Public Health, University of Colorado Anschutz Medical Campus, Box B119, 13001 E. 17th Place, Aurora, CO 80045 USA; 3 Colorado Cardiovascular Outcomes Research Consortium, Denver, 13611 E. Colfax Ave, A151, Aurora, CO 80045 USA; 4 Division of Cardiology, University of Colorado School of Medicine, Aurora, CO USA; 5 Division of Cardiology, Washington University School of Medicine, Campus Box 8086, 660 S. Euclid, St. Louis, MO 63110 USA

**Keywords:** Facility variation, Generalized linear mixed model, Hierarchical model, Hospital variation, Interval odds ratio, Median odds ratio, Random effect

## Abstract

**Background:**

Multilevel models for non-normal outcomes are widely used in medical and health sciences research. While methods for interpreting fixed effects are well-developed, methods to quantify and interpret random cluster variation and compare it with other sources of variation are less established. Random cluster variation, sometimes referred to as general contextual effects (GCE), may be the main focus of a study; therefore, easily interpretable methods are needed to quantify GCE. We propose a Reference Effect Measure (REM) approach to 1) quantify GCE and compare it to individual subject and cluster covariate effects, and 2) quantify relative magnitudes of GCE and variation from sets of measured factors.

**Methods:**

To illustrate REM, we consider a two-level mixed logistic model with patients clustered within hospitals and a random intercept for hospitals. We compare patients at hospitals at given percentiles of the estimated random effect distribution to patients at a median or ‘reference’ hospital. These estimates are then compared numerically and graphically to individual fixed effects to quantify GCE in the context of effects of other measured variables (aim 1). We then extend this approach by comparing variation from the random effect distribution to variation from sets of fixed effects to understand their magnitudes relative to overall outcome variation (aim 2).

**Results:**

Using an example of initiation of rhythm control treatment in atrial fibrillation (AF) patients within the Veterans Affairs (VA), we use REM to demonstrate that random variation across hospitals (GCE) in initiation of treatment is substantially greater than that due to most individual patient factors, and explains at least as much variation in treatment initiation as do all patient factors combined. These results are contrasted with a relatively small GCE compared with patient factors in 1 year mortality following hospitalization for AF patients.

**Conclusions:**

REM provides a means of quantifying random effect variation (GCE) with multilevel data and can be used to explore drivers of outcome variation. This method is easily interpretable and can be presented visually. REM offers a simple, interpretable approach for evaluating questions of growing importance in the study of health care systems.

## Background

Multilevel or hierarchical designs and clustered data are now commonplace in many fields including medical and health sciences research. These designs involve units within clusters, for example patients or subjects clustered at sites or centers, or multiple measurements of an outcome clustered on individual subjects. Models for multilevel data may contain fixed effects describing measured characteristics at any level of the design, as well as random effects describing unexplained cluster variation. Non-normal outcomes such as binary, skewed, count, or time to event outcomes are also common and present additional challenges due to non-linearity, different scales (e.g. probability, rate, or hazard), and different effect measures (e.g. risk ratio, odds ratio, rate ratio, and hazard ratio).

One very common situation in health services research involves patients clustered within sites. In these situations, fixed effects are constructed for measured patient risks and site characteristics, and random effects are used to model unexplained variation in outcomes or procedure use across sites. This unexplained variation, sometimes referred to as general contextual effects (GCE) [1, 2], induces dependence among subjects within a cluster and so inference for fixed effects requires analytic methods that can accommodate this dependence such as generalized linear mixed models (GLMM), multilevel regression models (MLRM), or generalized estimating equations (GEE) [3–7]. Methods for estimation and inference for fixed effects in these models are well established and described in many references e.g. [3–6]. These analyses and results are standard for clinical studies and answer one set of important questions, estimation and inference for associations between individual fixed effects and outcomes.

Several other objectives in the analysis of multilevel designs are less often recognized, and methods to study them are less well developed. First, GCE is often important in its own right and can be the main focus of a study because it represents unexplained differences across treatment sites in clinical outcomes, cost, use of procedures or compliance with guidelines. Second, quantifying variation due to sets of covariates, such as all patient risks or all hospital characteristics, in comparison to GCE is also important to understand the relative contribution of each. Some work has been done in comparing sets of risks or characteristics to GCE to help place both in context, e.g. [1, 8], but more attention is needed for this important problem. In this paper we propose methods for studying these two objectives.

While most previous studies have considered GCE when assessing clinical outcomes [9–13], the same analytic methods can be used to study GCE in processes of care [14], as for example in [1] who have studied use of medications and specialty physicians. In these studies, GCE is particularly important for several reasons. First, GCE in health care processes represents variation that is not driven by patient characteristics or treatment guidelines, since results are virtually always adjusted for patient risk. Second, variation of GCE in processes of care is potentially modifiable through site-specific interventions, which can be tested using trial designs such as cluster-randomized [15–18] or stepped-wedge [19] designs. Finally, GCE, particularly in processes of care, can be large, even dominating measured patient and hospital effects, as we illustrate in our application below.

Several categories of methods have been used to study and quantify GCE, each addressing different questions. One set of methods, exemplified by intra-class correlation (ICC) [20] and percent change in variance [1], seeks to determine the percent of total variation in outcomes due to cluster variation. These methods are useful in answering questions about relative sources of variation, but are not directly comparable to effect measures such as odds ratios. A second set of methods has the goal of quantifying GCE in comparison to fixed effects. One simple approach, which we call Individual Outcome Measures (IOM), involves calculating intervals for the mean outcome (e.g. probability) as the cluster random effect ranges across its (usually normal) distribution e.g. [3, 4]; however, interval widths differ for each covariate pattern making comparisons difficult and impractical with more than a few covariates. Additional approaches, such as Median and Interval Odds Ratios (MOR, IOR) [21–23] and more recently Median Hazard Ratios (MHR) [2], are based on odds or hazard ratios comparing subjects in two randomly selected clusters. A third set of methods studies individual clusters by ranking them or identifying outlying clusters, but is limited in that differential cluster size may lead to small clusters being more likely to have extreme averages. More sophisticated statistical methods, termed institutional profiling, are based on GLMM or MLRM and are used extensively in health services research [24]. These methods only indirectly answer the question of cluster variation since variability in cluster-specific estimates is affected by sample size. A recent paper has reviewed and combined some of these methods discussed above into a stepwise procedure [1].

We focus on methods that quantify and compare fixed and random effects on the effect (e.g. odds ratio) scale and so address questions similar to those of MOR/MHR. The methods we propose provide some advantages in terms of interpretation and visualization, as we discuss below. The approach we describe is based on comparing subjects in clusters at specified percentiles of the random effect distributions to subjects in a “reference”, e.g. median, cluster. We refer to these methods as Reference Effect Measures (REM) [25]. Subjects are compared based on the relevant effect measure, for example odds ratios for logistic regression. By comparing two subjects with identical covariate patterns the resulting odds ratio between subjects in two clusters does not depend on the particular covariate pattern. This approach is based on percentiles and so generalizes easily to non-normal or empirical distributions, and is amenable to graphical presentation.

To illustrate these methods, we use data from the Department of Veterans Affairs (VA) for a population of patients with an index inpatient admission for atrial fibrillation (AF), the most common form of cardiac arrhythmia, between 2001 and 2012 at one of the 124 VA hospitals that treat such patients. Typical studies within this setting adjust for and/or assess associations of 10–30 patient and hospital characteristics with binary, continuous, or time to event patient outcomes. Variation in these outcomes across hospitals is an inescapable feature of the data and is often of primary clinical or health services research interest. The example described in detail below involves mixed logistic regression models for probability of mortality and of initiation of a cardiac rhythm control strategy within 90 days after discharge from the index admission for AF.

In this paper we consider two objectives in the analysis of cluster variation for non-normal outcomes in multilevel designs: 1) Quantify GCE and compare it to individual subject and cluster covariate effects, and 2) Quantify relative magnitudes of GCE and variation from sets of measured factors. In the Methods section we first summarize concepts and notation for multilevel logistic models. We then describe REM methods in more detail and discuss how these methods address our two objectives. We also describe use of REM with other common situations such as GLMM and Cox proportional hazards models. In the Results section we apply these methods to objectives 1 and 2 for hospital variation in selection of treatments for atrial fibrillation patients at 124 VA hospitals. We also illustrate and compare several alternative methods. In a second example we contrast these results with hospital variation in mortality, and illustrate methods for visualizing and presenting results in easily interpretable ways.

## Methods

### Notation and models

To simplify presentation and notation we consider two-level designs with level 1 representing subjects and level 2 representing clusters, and consider the common case of mixed effects logistic regression for binary responses. For subject *j*, *j* = 1, …, *n*_*i*_ in cluster *i*,  *i* = 1, …, *N*, *Y*_*ij*_ is a binary outcome taking value 1 with probability *p*_*ij*_ and 0 otherwise. Let ***x***_*ij*_ be a vector of covariates that can be partitioned into a column of 1^′^s for the intercept, subject level covariates ***x***_*sij*_ and cluster level covariates ***x***_*cij*_, with corresponding parameter vectors ***β***, *β*_0,  _***β***_*s*,_ *and* ***β***_*c*_ . We use ***x***_*ij*_ for an observed value of the covariate vector and ***x*** for a generic value. Unexplained cluster variation (GCE) is usually described by independent normal random effects ${u}_i\sim \kern0.5em N\left(O,\kern0.5em {\sigma}_u^2\right)$, sometimes called random intercepts, although other distributional assumptions can be made. We let ϕ^−1^(*a*) denote the 100  × *a* percentile of the standard normal distribution. The mixed effect logistic regression model is.

1 $$ {Y}_{ij}\left|{p}_{ij}\kern0.5em \sim \kern0.5em Bin\left(1,\kern0.5em {p}_{ij}\right)\kern0.5em with\kern0.5em {p}_{ij}\kern0.5em =\kern0.5em \exp \kern0.5em \left({L}_{ij}\right)/\left(1\kern0.5em +\kern0.5em \exp \left({L}_{ij}\right)\right)\kern0.5em \right. $$

where *L*_*ij*_  =  ***x***'_***ij***_ ***β***  + *u*_*i*_ is the linear predictor.

For logistic regression the natural description of covariate effects is the odds ratio. The odds ratio (OR) for a subject with probability *p*^∗^ and linear predictor *L*^∗^ compared to a subject with probability *p* and linear predictor *L* is

2 $$ OR\left({L}^{\ast },\kern0.5em L\right)\kern0.5em =\frac{p^{\ast }/\left(1-{p}^{\ast}\right)}{p/\left(1-p\right)}\kern0.5em =\frac{\left[\mathit{\exp}\left({L}^{\ast}\right)\kern0.5em /\left(1+\mathit{\exp}\left({L}^{\ast}\right)\right)\right]/\left[1/\left(1+\mathit{\exp}\left({L}^{\ast}\right)\right)\right]}{\left[\mathit{\exp}(L)\kern0.5em /\left(1+\mathit{\exp}(L)\right)\right]/\left[1/\left(1+\mathit{\exp}(L)\right)\right]}=\frac{\mathit{\exp}\left({L}^{\ast}\right)}{\mathit{\exp}(L)}\kern0.5em =\mathit{\exp}\left({L}^{\ast }-L\right) $$

Measured subject and cluster covariates and unmeasured cluster random effects that are equal for the two subjects subtract out of this expression so the resulting odds ratio does not depend on their specific values. Thus, the odds ratio for a one unit difference in a single covariate *x*_*k*_, comparing subjects with all other measured subject and cluster covariates and unmeasured cluster random effects equal, is as usual *exp*(*β*_*k*_).

### Reference effect measures (REM)

#### Quantifying cluster variation (objective 1)

To describe GCE, REM uses measures based on comparison of patients at specified percentiles of the random effect distribution. A subject in a 100 × *α* percentile cluster compared to a subject in a median (50th percentile) cluster, with all measured subject and cluster covariates ***x*** equal, has *L*^∗^  =  ***x***^'^***β*** + *σ*_*u*_**ϕ**^**−1**^(*a*) *and L* = ***x***^'^***β*** + *σ*_*u*_ϕ^−1^(0.5) = ***x***^'^***β***, giving odds ratio

3 $$ {REM}_u(a)=\mathit{\exp}\left({\sigma}_u{\upphi}^{-1}(a)\right) $$

A 95% range of such odds ratios is [*exp*(−1.96*σ*_*u*_),   *exp* (1.96*σ*_*u*_)]. This range is not a confidence interval, but rather describes unexplained cluster variation in terms of odds ratios relative to a median cluster, as clusters range across their distribution. The 95% REM range can be thought of as analogous to the ‘95% rule’ for normal distributions, enclosing the middle 95% of the distribution, but expressed on the effect rather than linear predictor scale. We use square brackets [⋅] to denote REM ranges and round brackets (⋅) to denote confidence intervals. In applications *σ*_*u*_ is replaced by its estimated value ${\widehat{\sigma}}_u$**.**

We can also use REM to compare the effect of a 1 unit increase in a covariate *x*_*k*_ to unmeasured cluster variation by calculating $\upphi \left(\frac{\beta_k}{\sigma_u}\right)$, which provides the equivalent percentile in the random effects distribution. Thus, the effect of a 1 unit increase in *x*_*k*_ is the same as comparing a subject in a $100\times \upphi \kern0.5em \left(\frac{\beta_k}{\sigma_u}\right)$ percentile cluster with the same subject at a median risk cluster. Similar approaches to REM have been used by several authors including Spiegelhalter et al. [26] and Timbie et al. [27] to describe prior distributions in Bayesian analyses, and by Lingsma et al. [28] in studying heterogeneity of treatment effect across clusters, but apparently have not been widely used or developed.

#### Quantifying variation from sets of covariates (objective 2)

While we can quantify the relative size of GCE by comparing it to the effects of single covariates using REM as above, treatment decisions and guidelines are often driven by multiple factors; thus, comparisons to effects of combinations of factors may be of interest. To determine the magnitude of associations of sets of measured factors with outcomes, REM methods can also be applied to the empirical distribution of observed values from a set of covariates. For simplicity we describe methods for the empirical distribution of measured subject factors {***x***'_***sij***_***β***_***s***_} although these methods can easily be applied to the full set of covariates {***x***'_***ij***_***β***}, measured cluster factors {***x***'_***cij***_***β***_***c***_}, or other subsets of covariates. In applications ***β***^'^*s* are replaced by their estimates.

Variation explained by a set of covariates *x*_*s*_ can be quantified by calculating the empirical distribution of odds ratios for each subject compared with a subject of median risk within the empirical distribution of {***x***'_***sij***_***β***_***s***_}, all in a cluster with the same unmeasured characteristics *u* and holding constant all other measured characteristics, designated by ***x***_−***s***_. The expression compares odds ratios for ${L}^{\ast }={F}_{\left\{x{\hbox{'}}_{sij}{\beta}_s\right\}}^{-1}(a)+u$ and $L={F}_{\left\{x{\hbox{'}}_{sij}{\beta}_s\right\}}^{-1}(0.5)+u$ where ${F}_{\left\{x{\hbox{'}}_{sij}{\beta}_s\right\}}^{-1}(a)$ is the 100 × *α* percentile of the empirical distribution of {*x*'_*sij*_*β*_*s*_}. By centering the empirical distribution at its median (i.e. comparing with a median risk subject), the resulting odds ratio is

4 $$ {REM}_{\left\{x{\hbox{'}}_{sij}{\beta}_s\right\}}(a)=\mathit{\exp}\left({F}_{\left\{x{\hbox{'}}_{sij}{\beta}_s\right\}}^{-1}(a)\right) $$

and a 95% range enclosing the middle 95% of the distribution of such odds ratios is $\left[\mathit{\exp}\left({F}_{\left\{x{\hbox{'}}_{sij}{\beta}_s\right\}}^{-1}(0.025)\right),\mathit{\exp}\left({F}_{\left\{x{\hbox{'}}_{sij}{\beta}_s\right\}}^{-1}(0.975)\right)\right]$. Wider ranges imply larger contributions to overall variation in the outcome.

Benefits of the REM approach include expression on the odds ratio scale, and accommodation of non-normal random effect or empirical distributions through use of percentiles, all of which facilitate numerical and graphical comparisons between all sets of fixed and random factors considered. These features are illustrated in the examples below. Silber et al. [8] proposed a similar approach based on empirical distributions like {***x***'_***sij***_***β***_***s***_}. However, they presented their results as ratios of variances of these distributions, analogous to ICC which differs from our approach of summarizing the percentiles of a distribution on the effect scale.

#### Confidence intervals

As with most statistical estimates, it is important to show the degree of uncertainty in the estimate, for example with confidence intervals (CI). Using most statistical software, one can extract standard errors $SE\left({\widehat{\sigma}}_u^2\right)$ or $SE\left({\widehat{\sigma}}_u\right)$, usually calculated by the delta method, which can then be used to calculate CIs for functions of ${\sigma}_u^2$ or *σ*_*u*_. For example, a 95% CI for *REM*_*u*_(*α*)is

$$ \left(\mathit{\exp}\left(\left({\widehat{\sigma}}_u-1.96 SE\right)\left({\widehat{\sigma}}_u\right)\right){\Phi}^{-1}\left(\alpha \right)\right),\mathit{\exp}\left(\left({\widehat{\sigma}}_u+1.96 SE\left({\widehat{\sigma}}_u\right)\right){\Phi}^{-1}\left(\alpha \right)\right). $$

It should be noted that distributions of variances are often skewed and thus, closed form solutions and Wald-type intervals may not be appropriate. Confidence intervals can also be obtained by bootstrap, which also provides confidence intervals for measures such as *REM*{***x***'_***sij***_***β***_***s***_}(*a*) involving empirical distributions for combined risks or for percentiles corresponding to a specific REM. Due to the hierarchical nature of the analysis, bootstrapping methods that incorporate a multi-level structure should be used [29]. Such intervals are illustrated in the examples below. If models are estimated by Bayesian Markov chain Monte Carlo (MCMC), highest density credibility intervals (CrI) can be obtained from the MCMC samples. These also incorporate uncertainty in all estimated parameters.

### Extensions to other models and non-normal random effects

The methods described above apply equally well to other types of outcomes and models, for example generalized linear mixed models for normal, Gamma or Poisson outcomes e.g. [3, 4], or Cox proportional hazard frailty models for time to event outcomes [30–32]. The derivations and results above require the obvious modifications, and results are expressed on the corresponding scale, e.g. mean differences for normal models, rate ratios for Poisson models, or hazard ratios for Cox models. Extensions can also be made to models with more complex random effects, such as random slope or random coefficient models e.g. [28, 33].

In some situations, the normal distribution for the random effects *u*_*i*_ is replaced with another form. For example, log-Gamma random effects are often used in models based on Poisson distributions, because the resulting marginal distribution integrated over the random effects *f*(*y*) =  ∫ *f*(*y*|*u*)*g*(*u*)*du* can be calculated in closed form to be negative binomial [34]. Using REM methods with non-normal random effect distributions is straightforward, replacing the term *σ*_*u*_ϕ^−1^(*a*) in (3) with percentiles from the specified random effect distribution.

### Example 1: Dataset and statistical analysis

Atrial fibrillation (AF) is the most common form of cardiac arrhythmia, affecting an estimated 2.2 million Americans [35]. If left untreated AF can lead to stroke and heart failure [36]. Management options include anticoagulation to prevent blood clots leading to stroke, rate control to reduce heart rate, and rhythm control to return heart rhythm to normal using antiarrhythmic medications or cardioversion using electricity or drugs. We examined a population of 29,759 patients with an index inpatient admission for AF at 124 hospitals within the VA health system between October 2001 and September 2012 for initiation of rhythm control treatment as identified by a prescription for an antiarrhythmic drug (Procainamide, Quinidine, Disopyramide, Mexiletine, Propafenone, Flecainide, Amiodarone, Sotatlol, Dofetilide, or Dronedarone) or cardioversion on multiple days following hospitalization. Of the 29,759 patients, 4335 (14.6%) were started on an antiarrhythmic medication within 90 days after discharge from their index AF admission. Although treatment patterns for similar patients should remain consistent across hospitals due to common guideline recommendations, we hypothesized that rhythm control strategy may differ by site even after accounting for measured patient and hospital characteristics. Therefore, we would like to quantify the extent of GCE, our objective 1. Furthermore, the decision for rhythm control should ideally be directed by multiple patient factors. Thus, it is of interest to investigate the extent to which patient characteristics are a driving factor in the initiation of rhythm control treatment relative to measured hospital characteristics and unmeasured hospital variation, our objective 2.

To perform this analysis for Example 1, the outcome variable was dichotomized as initiation of rhythm control medications (y/n) within 90 days after hospital discharge from the index AF admission, and all patients were followed for at least 90 days. We used mixed logistic regression including multiple patient factors (age, sex, race, rurality, prior rate control strategy based on a previous prescription for a beta-blocker, calcium channel blocker of Digoxin, prior anticoagulant (AC), prior antiplatelet, CHA2DS2-VASc score, alcohol use, chronic kidney disease (CKD), congestive heart failure (CHF), depression, diabetes, drug abuse, liver disease, peripheral artery disease (PAD), prior myocardial infarction (MI), prior stroke, sleep apnea, time trend) and site factors (region of U.S., electrophysiology (EP) care onsite, site volume, academic affiliation, rural proportion). A site random normal intercept was included to incorporate unmeasured site variation. A non-linear relationship was found to exist between calendar time and rhythm control use so calendar time was included as a cubic spline. We provide results from this small set of covariates for illustration of the methods discussed in this paper but do not consider it a definitive clinical analysis, which requires more covariates and will be published elsewhere. All analyses were carried out using R software [37]. Parameters were estimated using maximum likelihood with the ‘glmmML’ package in R version 3.2.5 [38], but REM methods would apply equally well to parameters estimated using MCMC, for example in the WinBUGS software [39]. R code for the examples is available from the authors upon request.

### Example 2: Dataset and statistical analysis

Our second example illustrates tabular presentation of REM and different methods for calculating CIs, as well as a different pattern of variation. In the same cohort of AF patients, we examined factors contributing to variation in 1-year all cause mortality, where 14.9% of patients died within 1 year following discharge from their index AF admission. We again used logistic regression, with a binary outcome for 1-year mortality and the same patient and site characteristics as in Example 1.

## Results

### Example 1

Standard logistic regression results are given in Table 1, and show that several patient risk factors are strongly associated with use of rhythm control treatment (*p* <  0.001), but no measured hospital factors are significantly associated with rhythm control.

**Table 1:** Example 1 Model Results

	Estimate	SE	*P*-value	OR	95% CI for OR
(Intercept)	−2.292	0.321	< 0.001	0.101	(0.054, 0.190)
Patient Risk					
Alcohol Use	−0.358	0.061	< 0.001	0.699	(0.620, 0.788)
Liver Disease	−0.309	0.100	0.002	0.734	(0.604, 0.892)
Age (10 Years)	−0.267	0.017	< 0.001	0.766	(0.741, 0.791)
Drug Abuse	−0.202	0.098	0.039	0.817	(0.675, 0.990)
…					
White	0.342	0.055	< 0.001	1.407	(1.263, 1.568)
Prior AC	0.368	0.070	< 0.001	1.445	(1.261, 1.656)
CHF	0.409	0.039	< 0.001	1.505	(1.395, 1.623)
Hospital Risk					
Rural Prop.	−0.014	0.024	0.569	0.986	(0.941, 1.034)
…					
EP Onsite	0.084	0.127	0.509	1.088	(0.848, 1.395)
Hospital SD	0.511	0.040			

Standard logistic regression output for initiation of rhythm control treatment (y/n) for 29,759 AF patients at 124 VA hospitals during the period October 1, 2001 to September 30, 2012. Some covariates included in the model have been omitted from the table and figures to simplify presentation

From the model results ${\widehat{\sigma}}_u=0.511$ and a 95% REM range for odds ratios comparing patients at 2.5 and 97.5 percentile hospitals with patients at a median hospital is, from (3), [0.367, 2.772]. This wide range of odds ratios indicates substantial variation in rhythm control use across sites. For comparison, the odds of initiation of rhythm control are 1.50 times higher for a congestive heart failure (CHF) patient relative to the same patient without CHF, the largest individual patient risk effect. This is equivalent to comparing a patient at a 79th percentile risk hospital (95% CI: (74, 86)) to a similar patient at a median risk hospital. Thus, the risk of being at the 21% highest risk hospitals (95% CI: (14, 26%)) compared with a median risk hospital exceeds that of the largest patient risk factor. These comparisons of GCE to individual patient characteristics further exhibit the large variation across hospitals in AF treatment decisions.

**Fig. 1:**
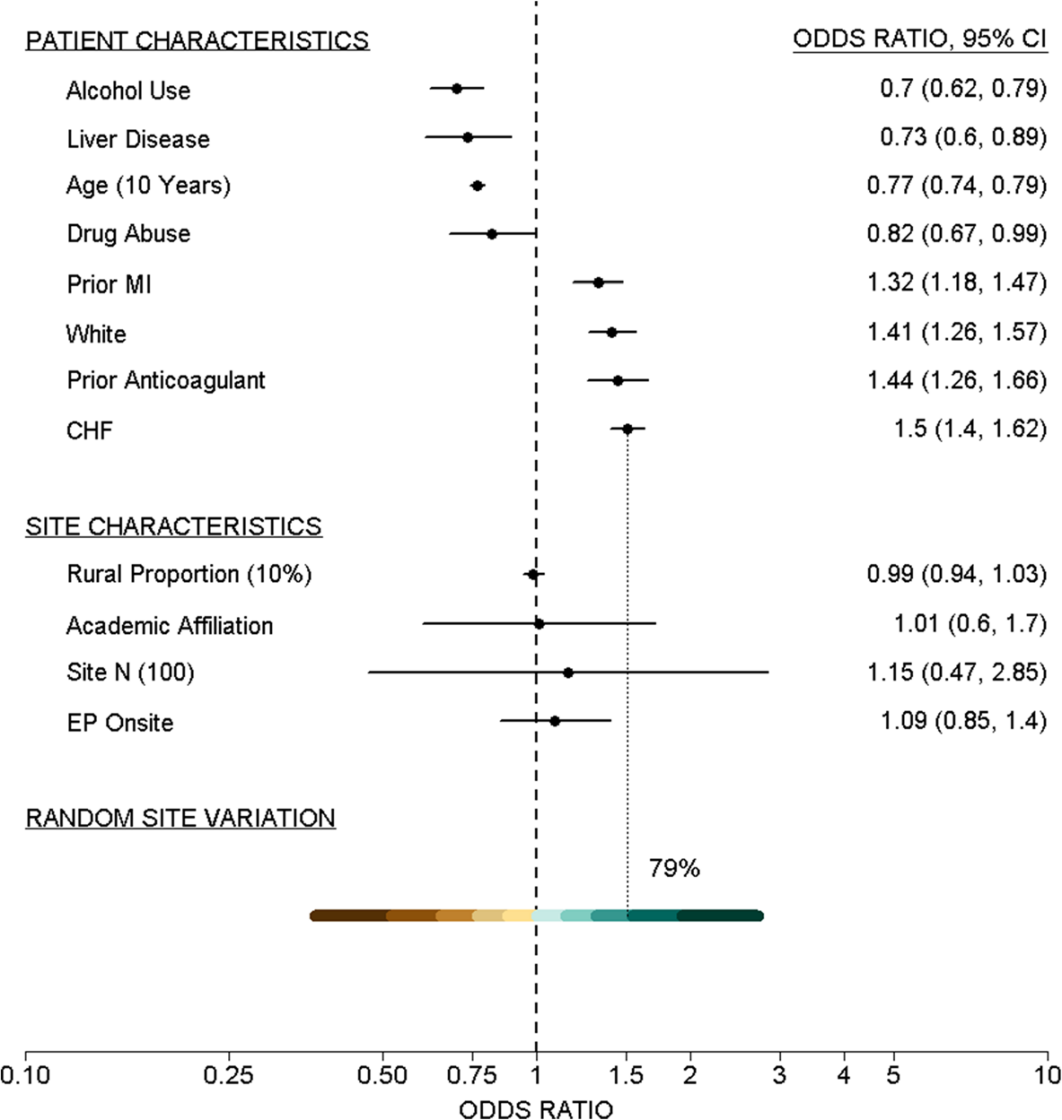
Example 1 Forest Plot. Forest plot showing odds ratios and 95% CIs for individual patient and site fixed effects, and REM ranges for unexplained hospital variation in use of rhythm control treatment for AF patients. Levels of shading represent 97.5, 90, 80, 70, 60, 50 percentiles (and corresponding lower percentiles)

An advantage of the REM approach is the ability to visualize the extent of GCE by plotting its distribution in a forest plot as shown in Fig. 1. A forest plot with the effect estimates and 95% CIs for other individual patient and site factors can be added to the plot. This allows direct comparisons between GCE and individual factors to quantify the extent of variation in the context of the analysis results.

**Fig. 2:**
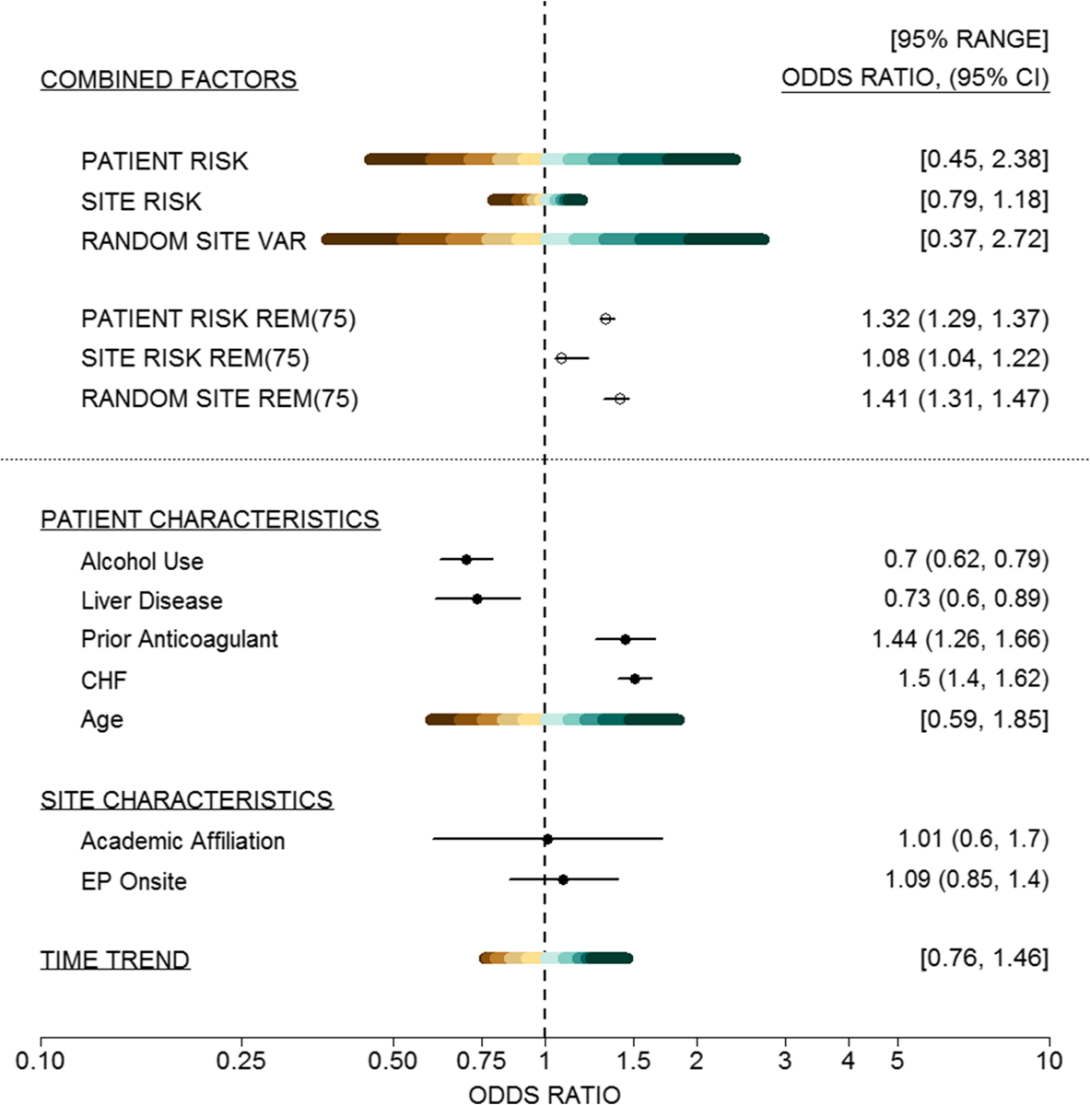
Example 1 REM Plot. 95% REM ranges and *REM*(0.75) for all patient risks, site characteristics and unmeasured site variation. Also indicated are individual risk effects and 95% REM ranges for age and time trend. Levels of shading represent 97.5, 90, 80, 70, 60, 50 percentiles (and corresponding lower percentiles)

The decision to use rhythm control would in practice be directed by multiple patient factors so we also used REM methods to explore the effects of combinations of factors. Using the previously described methods in (4), 95% REM ranges of odds ratios for all measured patient factors, all measured site factors, and GCE are [0.45, 2.38], [0.79, 1.18], and [0.37, 2.72] respectively, and are shown in Fig. 2. The wider ranges for combined patient factors and random site variation indicate that they contribute more to variation in the outcome and thus are larger drivers of rhythm control initiation. Precision of REM values can be given with 95% CIs. For example, we could present REM values comparing a given percentile risk, say 75th percentile, versus median risk. These values with 95% bootstrap CIs are 1.32 (1.29, 1.37) for measured patient factors, 1.08 (1.04, 1.22) for measured hospital factors, and 1.41 (1.31, 1.47) for unexplained site variation. The similar REM range widths and *REM*(0.75) values for measured patient factors and unmeasured site variation are interesting in their own right. Because we expect patient factors to largely drive treatment decisions, the fact that as much or more variation is driven by GCE highlights the need to further study reasons for these unexplained site differences, and possibly to consider studies and interventions to standardize treatment patterns across sites.

Once again, an advantage of the REM approach is the ability to visualize sources of variation as illustrated in Fig. 2. Investigators can visually compare the extent of variation driven from the different sources based on the widths of the REM distributions and ranges at the top of Fig. 2, while also making comparisons to individual factors. Different REM values can also be plotted as points with 95% CIs as shown. REM methods can also be used to display the risk distribution for continuous variables, e.g. age, rather than a point estimate and CI for a specified unit change in age (e.g. per year). Also, the risk distribution for non-linear variables, such as the cubic spline for calendar time, can be presented on the same plot where typically presentation of non-linear model results is limited to a separate plot of the actual spline function.

#### Comparison to other methods

Several other approaches are available to quantify unexplained site variation. Individual outcome measures (IOM) calculate the probability of the outcome for a specific covariate pattern and assess how this probability varies as the random cluster effect varies across its distribution e.g. [3, 4]. Thus, a 95% IOM interval calculates

5 $$ {IOM}_u\left(x,a\right)=\mathit{\exp}\left({x}^{\hbox{'}}\beta +{\sigma}_u{\upphi}^{-1}(a)\right)/\left(1+\mathit{\exp}\left({x}^{\hbox{'}}\beta +{\sigma}_u{\upphi}^{-1}(a)\right)\right) $$

for *α* = 0.025 and *α* = 0.975 for a specified subject and cluster covariate pattern ***x***. For rhythm control treatment, using a simple example of a median age patient with CHF and 0 for all other covariates, the probability of initiation of rhythm control is 13.3% with a 95% range of [5.3, 29.5%] across sites. While this 95% range provides a way of quantifying variation across sites on the probability scale, the width of the range is dependent on the patient covariate pattern, and with numerous covariates in the model such an approach is impractical. Also, it does not describe site variation on the same odds ratio scale as for fixed effects, making comparisons difficult.

The MOR approach is similar to REM in concept while differing in its interpretation. The basis for MOR is that the median of the distribution of odds ratios comparing two randomly selected subjects with the same covariate patterns, but in different clusters, comparing the higher risk subject to the lower risk subject, is equal to $\mathit{\exp}\left[\sqrt{2}{\sigma}_u{\upphi}^{-1}(0.75)\right]$. In the rhythm control example, the MOR is 1.63, which implies extensive site variation since this odds ratio exceeds the odds ratio of the largest patient risk factor, CHF. Like REM, MOR compares patients with the same covariate patterns but does not depend on the specific covariate pattern, and the result is on the same odds ratio scale as are fixed effects. However, the description of a distribution using comparisons between randomly selected subjects is not commonplace and may be hard to relate to the normal distribution specified in the model. Furthermore, MOR is based on the distribution of differences, making visualization on figures like those above less interpretable. We should note that MOR can be expressed as ${REM}_u\left(\upphi \left(\sqrt{2}{\upphi}^{-1}(0.75)\right)\right)={REM}_u(0.83)$, which may aid in its interpretability. The concept behind MOR could also be extended to assessing groups of measured factors by estimating the median of the empirical distribution of differences comparing the higher risk to lower patient patients in the same cluster using resampling; however, these results will still be subject to the same interpretability issues**.**

Another approach notes that the odds ratio comparing two subjects in clusters that differ by one SD of the random effect distribution, assuming equal subject and cluster covariates ***x***, can be found from (2) with *L*^∗^ = ***x***^'^***β*** + (*u* + *σ*_*u*_) *and L* = ***x***^'^***β*** + *u*,  *giving PerSD*_*u*_ =  *exp* (*σ*_*u*_). This can also be expressed as *REM*_*u*_(ϕ(1)) = *REM*_*u*_(0.84). This compares subjects in different clusters but with identical fixed effects, and is directly comparable to odds ratios for fixed effects, so can be included in tables and figures as such. For rhythm control, *PerSD*_*u*_ = 1.67, compared with the odds ratio for one SD younger age, *PerSD*_*age*_ = 1.36. This approach could also be extended to sets of covariates, using the SD of the empirical distribution. The most direct comparison for continuous covariates is with a per-SD change in the covariate, though for covariates or sets of covariates with non-normal distributions such comparisons could be misleading.

Finally, we note the relation between REM and ICC, since the latter is often used to describe cluster variation, particularly in sample size estimation. For logistic regression one standard definition of ICC is $$ ICC=\frac{\sigma_u^2}{\sigma_u^2+{\pi}^2/3} $$, where the$$ \pi \raisebox{1ex}{$2$}\!\left/ \!\raisebox{-1ex}{$3$}\right. $$ term is derived assuming a logistic GLMM as in (1) with latent subject level errors *e*_*ij*_ following a logistic distribution e.g. [6, 40]. For Example 1, *ICC* = 0.074, which would appear to indicate a small amount of the total variation in use of rhythm control treatment is due to GCE. In contrast, use of REM to quantify GCE shows cluster variation in treatment use to be significant and clinically large, illustrating the value of using REM to quantify GCE on the odds ratio scale. We also note that this definition of ICC is based on a particular assumed error structure, and that a number of other definitions of ICC have been proposed for binary outcomes [20, 40], including definitions on the probability rather than logistic scale, which can give very different values of ICC ([40], Table 1]).

### Example 2

To assess the extent of GCE in 1 year mortality for AF patients, *REM*_*u*_(0.975) is 1.29 (95% CI: (1.11, 1.35)). The effects of several individual patient factors, such as CKD (OR: 1.69, 95% CI: (1.55, 1.84)), Age (OR per decade: 1.72, 95% CI: (1.66, 1.78)), and Liver Disease (OR: 1.80, 95% CI: (1.53, 2.13)) exceed even the extremes of the distribution of unmeasured site variation. Table 2 gives an example of tabular presentation of REM results for measured patient factors, measured site factors and GCE. We describe and illustrate several ways of presenting REM results including *REM*(0.75) for moderate comparisons, *REM*(0.975) for extreme comparisons, and 95% ranges that enclose 95% of the distribution of odds ratios compared with the median to describe the variation in risk of mortality patients may face based on opposite extremes in the distribution of GCE or a set of factors. Wider ranges illustrate greater variation in mortality driven by GCE or the set of factors and thus, larger influence on the outcome. As before, REM values and confidence intervals can also be given to correspond to the effect of a particular explanatory variable. In contrast to variation in procedure use in Example 1, the much wider REM range for patient factors indicates that these are the largest drivers of mortality in this cohort. CIs for two values of REM based on theoretical standard errors from the delta method and from bootstrap distributions for *σ*_*u*_ indicate close agreement in this example. For comparison,*MOR* = 1.13 *and ICC* = 0.005, both also indicating little unexplained cluster variation.

**Table 2:** Example 2 REM Results

	95% Range	REM (0.75) and 95% CI	REM (0.975) and 95% CI
All patient risk factors	[0.23, 4.38]	1.69 (1.65, 1.74)	4.38 (4.12, 4.77)
All site characteristics	[0.81, 1.16]	1.06 (1.03, 1.11)	1.16 (1.09, 1.28)
Unmeasured site variation (theoretical)	[0.78, 1.29]	1.09 (1.06, 1.13)	1.29 (1.17, 1.41)
Unmeasured site variation (bootstrap)	[0.78, 1.29]	1.09, (1.04, 1.11)	1.29 (1.11, 1.35)

REM 95% ranges (in square brackets), and *REM*(0.75) and *REM*(0.975) with confidence intervals (in round brackets) for Example 2. Results are presented for all patient risk factors, all site characteristics and unmeasured site variation

## Discussion

In this paper we have proposed methods for quantifying and displaying variation in outcomes and treatments due to unexplained cluster sources as well as to sets of measured patient and hospital variables. These methods allow sources of variation to be studied on the same scale as effects of individual variables and, unlike other methods, can be easily incorporated into standard visual displays such as forest plots. Additionally, REM offers the flexibility of having standard values to report, such as *REM* (0.75) for moderate comparisons or *REM* (0.975) for extreme comparisons, while also allowing calculation of all other percentiles and a complete description of the distributions of interest. This is useful for directly relating the impact of a fixed effect to the exact percentile in the risk distribution of unmeasured site variation. Finally, the methods are widely applicable to multiple random effects or random slopes, empirical distributions, and random effects with non-normal distributions in multilevel studies. We used these methods to show that treatment for a common and serious cardiac condition (AF) is highly variable across VA hospitals, and this GCE is at least as great a source of variation in treatment use as all patient factors combined. These results suggest opportunities for study and improvement of patient care for AF patients, and illustrate the usefulness of the proposed methods.

As methods to model hierarchical data become more commonplace, it also becomes essential to develop meaningful and interpretable ways of presenting results. This is particularly true with random cluster variation (GCE), which has received much less attention than individual fixed effects, especially in the context of non-normal outcomes. With growing interest in studying processes of care and health care system-level questions, further fueled by growth of electronic health records (EHR), cluster variation will often be a factor of primary interest particularly for processes of care since these processes are often driven by unmeasured provider characteristics (e.g. preferences, training) or local culture that are difficult to capture in a model. Thus, understanding and explaining GCE in the context of other sources of variation will continue to become an area of focus moving forward.

Previously proposed methods for studying GCE are summarized in the Introduction and at the end of Example 1. Lack of a single summary measure for GCE is related to the fact that several questions can be asked about GCE. If the goal is to understand what proportion of total variation is attributed to cluster variation, ICC and related methods are available [1, 20]. If the goal is to rank or identify outlying sites, profiling methods can be used [24]. For quantifying GCE on the same scale as fixed effects, MOR methods have been used [21–23]. The recently proposed stepwise approach to analysis of variability for multilevel data considers several of these in their step 2 [1]. REM methods focus on quantifying GCE in the context of standard analyses through direct comparisons to individual fixed effects and sets of fixed effects on the same scale, similar to the questions addressed using MOR.

Interpretation of cluster level fixed effects has generated some controversy in the literature. Many authors interpret cluster level covariate effects in a similar way as patient level covariate effects, comparing patients with the same measured characteristics at hospitals with the same random and fixed effect values but differing by one unit in the cluster level covariate being considered. Others have argued that this interpretation is invalid since the design does not allow the same subjects to be observed at clusters with different cluster level covariate values. The latter interpretation motivated the development of the Interval Odds Ratio [21, 22]. We acknowledge the merit of the latter argument, but consider the former conditional interpretation valid in the context of the models used and the assumptions made. REM for cluster level covariates is consistent with the former interpretation, and with describing model (1) through components of the linear predictor *L* = *β*_0_ + ***x***'_***s***_***β***_***s***_ + ***x***'_***c***_***β***_***c***_ + *u*. We also note that this issue involves cluster level fixed effects but does not involve interpretation of subject level covariate effects or random cluster effects.

REM methods provide several advantages. The methods are general and apply to most types of outcomes and models commonly encountered in health research including binary, continuous, count, and time to event. The methods are based on percentiles, which allow more complete description of non-normal distributions that may arise from empirical distributions of sets of variables as in Eq. (4), or from use of non-normal random effects [34]. Percentiles also provide easy interpretations in terms of comparison with individual fixed effects and ranges or the ‘95% rule’ for normal distributions. These interpretations are more familiar than the interpretation of MOR, which summarizes a distribution of two patients at randomly selected clusters comparing the higher risk patient to the lower risk patient. A further advantage of REM is easy visualization, for example in forest plots like Fig. 1. When assessing factors driving variation in treatment use or mortality we presented ranges with shading showing several percentiles for different groupings of fixed effects and GCE. Widths of these ranges show visually which sets of measured factors and GCE are the largest drivers of variation in initiation of treatment. The distribution of differences between patients used to construct MOR is less interpretable graphically.

We have further explored the extent to which GCE drives variation in processes and outcomes through comparison of GCE to sets of fixed patient and hospital effects. Quantifying discriminatory ability of sets of fixed effects is also considered by [1] in their step 1, using area under the receiver operator curve (AUC). Our proposed REM approach for sets of fixed effects is complementary to AUC, and will be useful when direct comparisons with individual fixed effects or with GCE on the same scales are of most interest.

One issue we have not included in our analyses but that could be easily handled with REM involves imbalance of subject factors across clusters. This can occur when different hospitals tend to treat different types of patients. Methods are available for partitioning patient factors into between-cluster components (e.g. hospital averages) and within-cluster components (subject values or deviations of subject values from hospital averages) [41, 42]. Both between-cluster and within-cluster components can be included as covariates as usual, and REM methods would allow this component of variation to be quantified separately from patient factors and hospital system factors.

## Conclusions

REM provides a means of quantifying random effect variation (GCE) with multilevel data. The method is easily interpretable and can be presented visually, further assisting the reader in understanding results. This method also allows exploration of drivers of outcome variation including sets of fixed factors. Overall, while limited tools are available to quantify and compare these sources of variation, REM offers a simple, interpretable approach for evaluating questions of growing importance in clinical medicine and health services research.

## Abbreviations

AC: Anticoagulant
AF: Atrial Fibrillation
AUC: Area Under the Receiver Operator Curve
CHF: Congestive Heart Failure
CI: Confidence Interval
CKD: Chronic Kidney Disease
CrI: Credibility Intervals
EHR: Electronic Health Record
EP: Electrophysiology
GCE: General Contextual Effects
GEE: Generalized Estimating Equations
GLMM: Generalized Linear Mixed Models
ICC: Intra-Class Correlation
IOM: Individual Outcome Measures
IOR: Interval Odds Ratios
MCMC: Markov Chain Monte Carlo
MHR: Median Hazard Ratios
MI: Myocardial Infarction
MLRM: Multilevel Regression Models
MOR: Median Odds Ratios
OR: Odds Ratio
PAD: Peripheral Artery Disease
REM: Reference Effect Measure
SD: Standard Deviation
SE: Standard Error
VA: Veterans Affairs
